# Reporter Discrepancies in the Associations Between Mental Health Concerns and School Discipline

**DOI:** 10.1016/j.jaacop.2026.01.001

**Published:** 2026-01-13

**Authors:** Erin L. Thompson, Ashley R. Adams, Sarah M. Lehman, Christine Kaiver, Samuel W. Hawes, Kristin M. Scardamalia, Andy V. Pham, Raul Gonzalez

**Affiliations:** a Center for Children and Families, Florida International University, Miami, Florida; b Florida International University, Miami, Florida; c School of Education, University of Delaware, Newark, Delaware

**Keywords:** school discipline, adolescence, mental health, caregiver monitoring, ABCD Study

## Abstract

**Objective:**

The Adolescent Brain Cognitive Development℠ (ABCD) Study, which follows more than 11,000 adolescents, provides a unique opportunity to examine the causes and consequences of school detentions and suspensions. Although school records are considered the gold standard for assessing disciplinary events, they are not available in the ABCD Study®. Instead, youth and caregivers provide single-item reports of past-year discipline that may differ in scope and severity. The current study explored whether prospective associations with future school discipline varied by reporter, focusing on prior-year adolescent mental health concerns and discipline history as predictors.

**Method:**

Data from the ABCD Study (baseline n = 9,772; 8-11 years of age; 52% male) were analyzed using linear mixed-effects models with multiple imputation. Multi-informant mental health concerns (externalizing, inattention/impulsivity, and internalizing) from the first follow-up wave predicted discipline over the next 2 years across 4 discipline-reporting groups: youth-reported (20%), caregiver-reported (13%), agreement (10%), and either reporter (24%).

**Results:**

Youth-reported and either-reporter discipline measures showed higher prevalence rates but weaker bivariate associations with prior mental health and disciplinary history relative to caregiver-reported and agreement discipline measures; however, most differences diminished after covariate adjustment. Across reporters, prior discipline remained the strongest predictor of future discipline.

**Conclusion:**

Reporter choice in school discipline measurement influenced observed associations. Patterns suggested that youth-reported discipline may capture a broad range of infractions, whereas caregiver-reported discipline may reflect more severe events. However, recognizing the limited precision of single-item discipline indicators is essential for interpreting results and guiding future ABCD investigations.

## Plain language summary

This study evaluated how children’s mental health and past experiences with school discipline impacted their chances of getting disciplined again. Utilizing data from the Adolescent Brain Cognitive Development℠ (ABCD) Study (n = 9,772, aged 8-11 years), the authors found that while demographics can increase disciplinary risk, they do not fully account for discrepancies between youth and caregiver discipline reporting. This study also found that teacher-reported inattention/impulsivity showed the largest effect on subsequent discipline. Adolescents who received school discipline were at higher risk for repeated discipline independent of demographic characteristics and prior behavioral concerns. Consistent with prior studies, these results suggest that school discipline is not an effective strategy for reducing behavioral problems in schools.

School disciplinary practices (eg, detentions and suspensions) have drawn growing interest from educators, mental health providers, researchers, and policymakers. A large body of research demonstrates that youth with elevated behavioral and emotional symptoms, particularly externalizing and inattention/impulsivity concerns, are at higher risk for receiving school discipline.^1^, ^2^, ^3^, ^4^, ^5^ In response, many schools have adopted strategies to curb behavioral concerns associated with school discipline (eg, school-wide positive behavioral interventions, restorative practices), although inequities across race, sex, and income remain well documented.^5^, ^6^, ^7^, ^8^, ^9^, ^10^ These persistent disparities, along with evidence linking school discipline to adverse long-term outcomes,^4^^,^^6^, ^7^, ^8^ underscore the need for continued research on school discipline.

The Adolescent Brain Cognitive Development℠ (ABCD) Study,^9^ a 10-year longitudinal study of more than 11,000 adolescents across 17 US states, is uniquely positioned to examine novel associations between school discipline and a wide array of constructs associated with adolescent development, including environmental contexts, brain development, physical and mental health, substance use, peer and family relationships, as well as longer-term educational and vocational outcomes. A few prior studies using data from the ABCD Study® have leveraged its heterogeneity to do the following: (1) to highlight the disproportionate use of school discipline among Black youth^10^, ^11^, ^12^; (2) to examine state- and neighborhood-level influences on discipline risk^12^^,^^13^; and (3) to investigate the heightened risk of police contacts among adolescents with a history of school discipline.^11^

Because of the difficulty of collecting records across hundreds of districts, the ABCD Study does not have access to official school disciplinary data. School administrative records are considered the gold standard for assessing school discipline; however, they are not always accessible, particularly in large, multi-site studies. Consequently, the ABCD Study asks youth and their parents or legal guardians (hereafter referred to as caregivers) to report on past-year detentions or suspensions. Researchers can therefore define school discipline using youth-report, caregiver-report, a joint endorsement measure (ie, agreement across reporters), or a composite whereby either reporter endorsed discipline. However, existing ABCD studies examining school discipline^10^, ^11^, ^12^, ^13^ have not provided a rationale for selecting one reporter over another, leading to variability in operational definitions and the potential to overlook contextual nuances.

Although much of the literature has focused on identifying predictors and consequences of school discipline, less is known about whether these associations differ depending on who reports the disciplinary event. Differences between youth and caregiver reports likely reflect variation in construct coverage, such as the types or severities of infractions captured, rather than poor interrater reliability. However, these interpretations should be considered with caution, as the ABCD Study discipline measure combines detentions and suspensions and does not capture how often they occurred. From this perspective, examining reporter-based differences can serve as a form of sensitivity analysis, helping to determine whether conclusions about discipline risk are robust across plausible variations in measurement and to identify the unique contextual information that each source may contribute.

### Differences in Discipline Severity Across Reporters

Insights from related fields suggest that discrepancies between reports can be meaningful. Extensive research on mental health has demonstrated that reporter discrepancies reflect distinct perspectives or contexts rather than measurement error.^14^ For school discipline, differences in what each informant can observe or is informed about may similarly shape reporting. For example, caregivers may be unaware of lower-severity infractions if they are not notified,^15^ whereas youth are directly aware of all incidents, including minor in-school detentions. This difference in informational access, or coverage, could influence prevalence estimates and the apparent strength of associations with known risk factors. For example, grouping in-school detentions for tardiness with more severe actions such as out-of-school suspension for physical aggression may attenuate associations with prior behavioral concerns, whereas focusing on more severe incidents could yield stronger associations.^2^^,^^8^^,^^16^

It is also possible that differences in construct coverage may also affect longitudinal associations between repeated school discipline. Prior work^2^^,^^3^^,^^17^ suggests that using a broad measure of school discipline, as is likely with youth-reported discipline, could weaken associations between prior and future school discipline. For instance, one study^17^ found that more severe disciplinary experiences were more strongly associated with repeated infractions in subsequent school years. Therefore, potential differences in the type and severity of infractions captured by youth vs caregiver reporters may help explain discrepancies in how strongly prior discipline predicts future infractions. However, this interpretation remains speculative and warrants further investigation, as we are not aware of existing studies that directly compared these associations across youth- and caregiver-reported school discipline.

### Present Study

Based on prior research,^1^, ^2^, ^3^^,^^5^^,^^8^ the current study examined whether recent mental health concerns (externalizing, inattention/impulsivity, and internalizing concerns) and past-year school discipline differentially predicted subsequent school discipline over the following 2 years, based on 4 reporting sources: (1) youth-reported, (2) caregiver-reported, (3) agreement across reporters, and (4) either reporter. Although we could not directly determine what each school discipline measure captured, significant findings across reports could indicate meaningful variation in the type or severity of disciplinary experiences. To minimize misinterpretation from this variation, we focused primarily on factors already well established as strong predictors of school discipline.^1^, ^2^, ^3^^,^^5^^,^^6^

We hypothesized that, because of caregivers’ potential lack of awareness of minor infractions, youth would report higher rates of school discipline than caregivers (Hypothesis 1). Based on robust literature,^4^, ^5^, ^6^, ^7^ we expected that a history of mental health concerns and prior school discipline would predict increased risk for subsequent school discipline across all 4 reporting groups (Hypothesis 2). Given likely differences in construct coverage, we further expected stronger associations between externalizing and inattention/impulsivity concerns and school discipline for caregiver-reported and agreement across reporters than for youth-reported and either reporter groups (Hypothesis 3). Similarly, we expected prior school discipline to more strongly predict future school discipline using the caregiver-reported and agreement across reporters discipline measures (Hypothesis 4). Differential associations between internalizing concerns and school discipline were considered exploratory.

## Method

### Participants and Procedure

We used the first 4 waves of data (T0-T3) from the ABCD Study (Data Release 5.1) from the National Institute of Mental Health data archive. More than 11,000 children, primarily 9 and 10 years of age, were recruited between 2016 and 2018 across 21 US sites located in 17 states, with ongoing assessment across 10 years. Albeit a diverse sample, participants lived in higher cost-of-living urban areas with caregivers who had higher incomes and educational attainment compared to the overall US population of individuals 9 to 10 years of age. Each site’s institutional review board approved the ABCD protocol, and informed child assent and caregiver consent were obtained at each wave. Detailed information about the recruitment process is available elsewhere.^16^^,^^18^ Table S1, available online, provides more information on the sample’s demographic characteristics.

### Measures

#### School Discipline

At each wave, youth and caregivers were asked whether the youth had received a detention/suspension in the last year (yes/no). For this study, school discipline at T1 was used as a predictor. To capture risk for subsequent discipline, responses from T2 and T3 were combined into a single binary outcome, reflecting whether the youth had received at least one detention or suspension across the 2-year follow-up period. The number of disciplinary events was not assessed.

#### Mental Health Concerns

T1 externalizing, inattention/impulsivity, and internalizing concerns were assessed using the caregiver-reported Child Behavior Checklist^19^ raw scores and the youth- and teacher-reported Brief Problem Monitor mean scores.^20^ Prior work has shown acceptable to strong internal consistency (α = 0.72-0.97).^19^^,^^20^

#### Demographics and Potential Confounders

Based on prior ABCD studies examining risk of school discipline,^10^, ^11^, ^12^ we included the following demographics: sex, race/ethnicity, caregiver education, age, grade, family income, caregiver partner status (ie, household structure), school type (public, private, charter, other), and neighborhood opportunity, a nationally normed measure of neighborhood resources derived from the US census tracts between 2010 and 2015.^21^ We chose to include race and ethnicity as potential confounders because of the disproportionate use of school discipline among Black and Hispanic youth.^4^^,^^10^, ^11^, ^12^ Participant sex, race/ethnicity, household structure, caregiver education, and neighborhood resources were assessed at baseline (T0), whereas age, school grade, school type, and household income were assessed at T1.

### Data Analysis

To assist with model convergence, we chose one random participant from each family, resulting in a sample of 9,772 youth. Missingness was low to moderate (0%-15%), excluding teacher-reported data, which was significantly higher (>50%) (Table S1, available online). We then created 20 imputed datasets using the multivariate imputation by chained equations package for multilevel data in R,^22^ accounting for nesting within site. In addition to the constructs of interest and covariates, baseline youth- and caregiver-reported school discipline were also included as auxiliary variables within the imputation model. We then standardized all mental health concern variables to place them on a common scale and to facilitate interpretation, particularly given differences in original metrics (ie, raw vs mean scores) and high relative variability across measures.

We ran separate generalized linear mixed-effects models accounting for nesting within site (n = 9,722 for all analytic models). We first tested whether the strength of the bivariate associations between each mental health concern and school discipline, as well as between repeated discipline, significantly differed across reporters. We then ran follow-up models that added sociodemographic characteristics, T1 school discipline, and T1 comorbidities. Specifically, when examining the effect of caregiver-reported externalizing concerns on discipline, the model also included caregiver-reported inattention/impulsivity and internalizing concerns. Because youth-reported mental health concerns were not collected at T0, T1 measures were used as the primary predictors of interest. Because of multicollinearity between age and grade, as well as between household income and caregiver education, we excluded age and income from the final models. Effect sizes are provided as odds ratios (ORs).

To assess whether the strength of associations differed significantly across discipline outcome types, we conducted pairwise Wald *z* tests. These were calculated as the difference between 2 unstandardized beta coefficients divided by the square root of the sum of their squared standard errors. Because the outcomes were estimated in separate multiple-imputed models, cross-outcome covariances were not available and were therefore assumed to be zero. This independence assumption likely inflates standard errors, yielding conservative tests; accordingly, we did not apply additional multiple-comparisons adjustments, which is a noted potential limitation. This study was not preregistered. The publicly available data used may be found at https://doi.org/10.15154/z563-zd24.

## Results

After pooling across imputed datasets, 76% of youth–caregiver pairs reported no disciplinary action at T2 or T3. Consistent with H1, youth reported significantly higher rates of school discipline than caregivers (20% vs 13%, *p* < .001). Approximately 10% of youth–caregiver pairs both reported school discipline, whereas using an “either-reporter” approach resulted in a 24% prevalence rate (Table 1).

**Table 1:** Participant Demographics Across School Discipline Reporter Groups

Predictors	Outcomes				Comparisons^c^ (*p* < .05)
	A	B	C	D	
	Youth-reported discipline	Caregiver-reported discipline	Agreement across reporters	Either reporter	
Overall disciplinary rates, %	20.04 (0.40)	13.10 (0.34)	9.58 (0.30)	23.56 (0.43)	D > A > B > C
Grade, mean	5.35 (0.02)	5.30 (0.02)	5.33 (0.02)	5.33 (0.02)	None
Age, mean	10.83 (0.01)	10.81 (0.02)	10.84 (0.02)	10.82 (0.01)	None
Male^a^, %	62.82 (1.09)	67.24 (1.31)	66.46 (1.54)	63.80 (1.00)	B > A, D
Two-caregiver household, %	69.88 (1.04)	67.95 (1.30)	66.66 (1.54)	70.11 (0.95)	None
**Neighborhood opportunity, mean**					
Very low opportunity	28.93 (1.02)	27.93 (1.25)	29.43 (1.49)	28.17 (0.94)	None
Low opportunity	16.80 (0.85)	14.92 (1.00)	15.24 (1.17)	16.39 (0.77)	None
Medium opportunity	14.15 (0.79)	15.26 (1.00)	15.34 (1.18)	14.29 (0.73)	None
Higher opportunity	19.79 (0.90)	21.27 (1.14)	20.88 (1.33)	20.17 (0.84)	None
Very high opportunity	20.32 (0.91)	20.62 (1.13)	19.11 (1.28)	20.98 (0.85)	None
**School type, %**					
Attend public school	80.93 (0.89)	80.12 (1.12)	79.92 (1.31)	80.89 (0.82)	None
Attend private school	4.91 (0.49)	4.29 (0.57)	4.29 (0.66)	4.82 (0.45)	None
Attend charter school	10.59 (0.70)	11.07 (0.88)	11.82 (1.06)	10.35 (0.63)	None
Attend other type of school	3.59 (0.42)	4.52 (0.58)	3.96 (0.64)	3.95 (0.41)	None
**Race and ethnicity, %**					
Black, non-Hispanic	26.06 (0.99)	27.39 (1.25)	27.97 (1.47)	26.03 (0.91)	None
Multiracial Black	9.41 (0.66)	10.39 (0.85)	11.00 (1.02)	9.31 (0.61)	None
Hispanic, non-Black	21.31 (0.93)	17.88 (1.07)	18.69 (1.27)	20.46 (0.84)	A > B
Other, non-Hispanic/non-Black^b^	6.43 (0.55)	6.55 (0.69)	6.10 (0.78)	6.63 (0.52)	None
White, non-Hispanic/non-Black	36.79 (1.09)	37.79 (1.35)	36.24 (1.57)	37.57 (1.01)	None
**Caregiver education, %**					
Less than high school diploma	8.16 (0.62)	7.16 (0.72)	7.89 (0.88)	7.71 (0.56)	None
High school diploma/GED	17.92 (0.87)	18.78 (1.09)	18.38 (1.27)	18.22 (0.80)	None
Some college	37.82 (1.10)	38.92 (1.36)	39.88 (1.60)	37.59 (1.01)	None
Bachelor’s degree	21.26 (0.92)	20.96 (1.14)	20.89 (1.33)	21.24 (0.85)	None
Post-Bachelor’s degree	14.84 (0.80)	14.18 (0.98)	12.96 (1.10)	15.24 (0.75)	None
**Household income, %**					
Under $50,000	46.04 (1.13)	47.53 (1.40)	48.91 (1.63)	45.71 (1.04)	None
$50,000-$99,999	27.42 (1.01)	27.03 (1.24)	27.45 (1.46)	27.19 (0.93)	None
$100,000 and over	26.54 (1.00)	25.44 (1.22)	23.65 (1.39)	27.10 (0.93)	D > C

Note: N = 9,772. Data in parentheses are standard errors. All predictors were assessed at the time 1, except for sex, race/ethnicity, household structure, caregiver education, and neighborhood resources, which were assessed at baseline (time 0). Binary discipline outcomes variables reflected school discipline reported at either time 2 or time 3 (ie, across a 2-year follow-up). Estimates were calculated after conducting multiple imputation.

a Fewer than 10 caregivers endorsed “intersex-male.” These were recoded as male.

b The “Other (non-Hispanic/non-Black)” race and ethnicity category included youth whose caregiver identified them as American Indian/Native American, Alaska Native, Native Hawaiian, Guamanian, Samoan, Other Pacific Islander, Asian Indian, Chinese, Filipino, Japanese, Korean, Vietnamese, Other Asian, Other race, or as belonging to more than one race (excluding African American/Black).

c Differences between subgroups were tested using z tests for means (for continuous variables) and z tests for proportions (for categorical variables). Statistically significant differences indicate that the demographic characteristic varied across the compared discipline groups at *p* < .05.

### Demographic Differences in Reporting of School Discipline

Reporter-based differences in school discipline did not significantly vary by most demographic variables. For instance, among youth living in low-opportunity neighborhoods, youth were no more likely than their caregivers to report school discipline. Across all comparisons, only 3 significant demographic findings emerged: (1) among Hispanic adolescents, school discipline via youth-reported discipline was more prevalent than via caregiver-reported discipline (*p* = .015); (2) among male individuals, school discipline via caregiver-reported discipline was more prevalent than via youth-reported discipline and either reporter (*p* = .010 and *p* = .037, respectively); and (3) among individuals from households earning more than $100,000, school discipline via either reporter yielded higher rates than using the agreement across reporters group (*p* = .038). Table 1 provides full results.

### Externalizing Concerns and Subsequent School Discipline

Youth-, caregiver-, and teacher-reported externalizing concerns were associated with increased odds of subsequent school discipline across all reporter groups (ORs = 1.55-1.92, *p* values <.001) (Table S2, available online). However, as expected, associations differed by reporter of school discipline. For caregiver-reported externalizing concerns, associations were stronger for caregiver-reported discipline (*p* < .001), agreement across reporters (*p* = .002), and either reporter (*p* < .001) vs youth-reported discipline, and stronger for agreement across reporters than for either reporter (*p* = .028). For teacher-reported externalizing concerns, associations were also stronger for caregiver-reported discipline (*p* = .014), agreement across reporters (*p* = .033), and either reporter (*p* = .023) vs youth-reported discipline. For youth-reported externalizing concerns, the largest ORs occurred for agreement across reporters, but associations did not significantly vary by discipline reporter.

**Figure 1:**
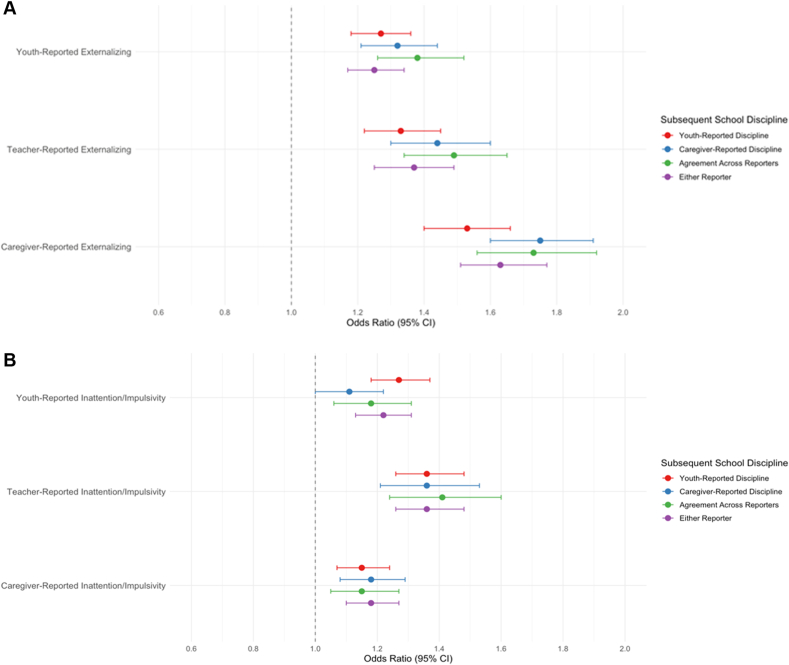

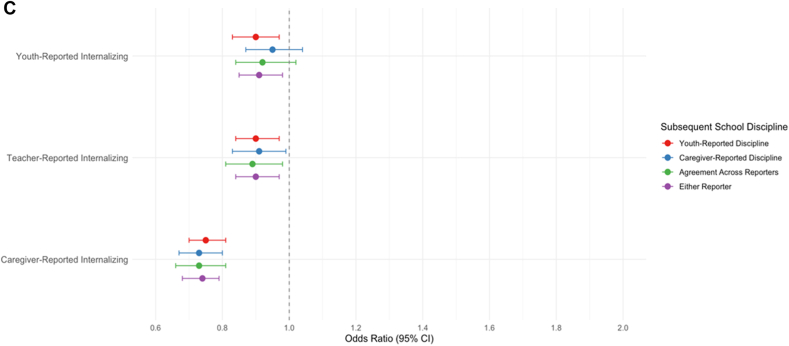
Associations Between Multirater Mental Health Concerns and Subsequent School Discipline (Covariate-Adjusted) ***Note:****(A) Externalizing concerns and subsequent school discipline. (B) Externalizing concerns and subsequent school discipline. (C) Internalizing concerns and subsequent school discipline.*

After adjusting for sociodemographics, prior discipline, and comorbidities, effect sizes attenuated (ORs = 1.25-1.75, *p* values <.001) (Table 2 and Figure 1A), indicating that a 1-SD increase in externalizing concerns was associated with a 25% to 75% increased odds of school discipline over the following 2 years, depending upon reporter. Caregiver-reported externalizing concerns remained more strongly associated with school discipline in the caregiver-reported discipline group than in the youth-reported discipline group (*p* = .032). Tables S3 to S5, available online, provide covariate effects.

**Table 2:** Covariate-Adjusted Associations Between Mental Health Concerns and School Discipline Across Reporters

Predictors	Outcomes												Comparison^a^*p* < .05
	A			B			C			D			
	Youth-reported discipline			Caregiver-reported discipline			Agreement across reporters			Either reporter			
	OR	95% CI	*p*	OR	95% CI	*p*	OR	95% CI	*p*	OR	95% CI	*p*	
**Externalizing Concerns**													
Youth-reported	1.27	[1.18, 1.36]	<.001	1.32	[1.21, 1.44]	<.001	1.38	[1.26, 1.52]	<.001	1.25	[1.17, 1.34]	<.001	None
Teacher-reported	1.33	[1.22, 1.45]	<.001	1.44	[1.30, 1.60]	<.001	1.49	[1.34, 1.65]	<.001	1.37	[1.25, 1.49]	<.001	None
Caregiver-reported	1.53	[1.40, 1.66]	<.001	1.75	[1.60, 1.91]	<.001	1.73	[1.56, 1.92]	<.001	1.63	[1.51, 1.77]	<.001	B > A
**Inattention/Impulsivity Concerns**													
Youth-reported	1.27	[1.18, 1.37]	<.001	1.11	[1.00, 1.22]	.049	1.18	[1.06, 1.31]	.002	1.22	[1.13, 1.31]	<.001	A > B
Teacher-reported	1.36	[1.26, 1.48]	<.001	1.36	[1.21, 1.53]	<.001	1.41	[1.24, 1.60]	<.001	1.36	[1.26, 1.48]	<.001	None
Caregiver-reported	1.15	[1.07, 1.24]	<.001	1.18	[1.08, 1.29]	<.001	1.15	[1.05, 1.27]	.003	1.18	[1.10, 1.27]	<.001	None
**Internalizing Concerns**													
Youth-reported	0.90	[0.83, 0.97]	.008	0.95	[0.87, 1.04]	.252	0.92	[0.84, 1.02]	.112	0.91	[0.84, 0.98]	.014	None
Teacher-reported	0.90	[0.84, 0.97]	.006	0.91	[0.83, 0.99]	.030	0.89	[0.81, 0.98]	.018	0.90	[0.84, 0.97]	.005	None
Caregiver-reported	0.75	[0.70, 0.81]	<.001	0.73	[0.67, 0.80]	<.001	0.73	[0.66, 0.81]	<.001	0.74	[0.68, 0.79]	<.001	None

Note: N = 9,772. Outcomes were measured as a collapsed binary variable indicating whether discipline occurred at either time 2 or time 3 (ie, during a 2-year follow-up period), whereas mental health concerns were assessed at time 1 (T1). Covariates included comorbid mental health concerns, past-year school discipline, race/ethnicity, sex, grade, caregiver education, household structure, school type, and neighborhood resources. OR = odds ratio.

a Comparisons reflect results from z tests evaluating whether the effect of a given predictor differed across regression models with different outcomes. Directional differences (eg, B > A) indicate a statistically significant difference in effect size at *p* < .05.

### Inattention/Impulsivity Concerns and Subsequent School Discipline

Youth-, caregiver-, and teacher-reported inattention/impulsivity concerns were each significantly associated with higher odds of subsequent school discipline across all reporter groups (ORs = 1.42-2.02, *p* values <.001) (Table S2, available online). For caregiver-reported inattention/impulsivity, associations were stronger for caregiver-reported discipline (*p* < .001), agreement across reporters (*p* = .004) and either reporter (*p* = .010) vs youth-reported discipline, and stronger for caregiver-reported discipline (*p* = .022) compared to either reporter. For teacher-reported inattention/impulsivity, associations were stronger for caregiver-reported discipline (*p* = .029) and agreement across reporters (*p* = .017) vs youth-reported discipline. Youth-reported inattention/impulsivity concerns associations were significant (ORs = 1.44-1.56, *p* values <.001) but did not significantly differ by discipline reporters.

After adjusting for sociodemographics, prior discipline, and comorbidities, effect sizes attenuated (ORs = 1.11-1.41, *p* values <.05) (Table 2 and Figure 1B), indicating that a 1-SD increase in inattention/impulsivity concerns was associated with a 11% to 41% increased odds of school discipline over the next 2 years. Associations between youth-reported inattention/impulsivity concerns and school discipline were stronger for youth-reported discipline than for caregiver-reported discipline (*p* = .026), which was not significant in the bivariate, unadjusted model. Sensitivity analyses indicated that this difference emerged only after controlling for other mental health concerns, not when controlling solely for sociodemographic characteristics. Tables S3 to S5, available online, provide covariate effects.

### Internalizing Concerns and Subsequent School Discipline

Youth-, caregiver-, and teacher-reported internalizing concerns were more modestly associated with subsequent school discipline (ORs = 1.08-1.30, *p* values <.003) (Table S2, available online). Caregiver-reported internalizing concerns were more strongly associated with school discipline when using caregiver-reported discipline (*p* = .004) and agreement across reporters (*p* = .029) vs youth-reported discipline, and stronger when using caregiver-reported discipline vs either reporter (*p* = .038). Teacher- and youth-reported internalizing concerns were not differentially associated with school discipline across reporters of discipline.

After adjusting for sociodemographics, prior discipline, and comorbidities, associations reversed in direction (ORs = 0.73-0.91, *p* values <.031) (Table 2 and Figure 1C), indicating that a 1-SD increase in internalizing concerns was associated with a 9% to 27% decreased odds of school discipline over the next 2 years. The only exceptions were non-significant associations between youth-reported internalizing concerns and both caregiver-reported discipline and agreement across reporters. Sensitivity analyses indicated that this reversal emerged only after including comorbid externalizing and inattention/impulsivity concerns, not when controlling solely for sociodemographics characteristics. No between-reporter differences remained statistically significant after covariate adjustment. Tables S3 to S5, available online, provide covariate effects.

### Risk for Repeated School Discipline

We next examined whether the association between prior discipline history and subsequent school discipline varied by reporter (Table 3). Prior school discipline was a robust predictor of receiving additional discipline over the next 2 years across all reporter groups (ORs = 6.36-9.86, *p* values <.001). As hypothesized, adolescents with a history of discipline were at greater risk for repeated discipline when it was measured via caregiver-reported discipline (*p* = .004) and agreement across reporters (*p* = .002), compared to measuring discipline via the youth-reported discipline, as well as stronger for caregiver-reported discipline (*p* = .039) and agreement across reporters (*p* = .016), compared to measuring discipline via either reporter.

**Table 3:** Risk of Repeated School Discipline by Reporter(s): Unadjusted Models vs Covariate-Adjusted Models

Model	OR	95% CI	*p*	Comparisons (*p*)		
				Caregiver-reported discipline T1 → T2/T3	Agreement across reporters T1 → T2/T3	Either reporter T1 → T2/T3
**Unadjusted models (no covariates)**						
Youth-reported discipline T1 → T2/T3	6.36	[5.48, 7.38]	<.001	.004	.002	.145
Caregiver-reported discipline T1 → T2/T3	9.05	[7.52, 10.90]	<.001	—	.571	.039
Agreement across reporters T1 → T2/T3	9.86	[7.83, 12.42]	<.001	—	—	.016
Either reporter T1 → T2/T3	7.11	[6.21, 8.13]	<.001	—	—	—
**Adjusted models (with covariates, including caregiver-reported mental health concerns)**						
Youth-reported discipline T1 → T2/T3	3.74	[3.17, 4.40]	<.001	.461	.540	.621
Caregiver-reported discipline T1 → T2/T3	4.10	[3.31, 5.06]	<.001	—	.988	.737
Agreement across reporters T1 → T2/T3	4.10	[3.13, 5.36]	<.001	—	—	.789
Either reporter T1 → T2/T3	3.94	[3.39, 4.58]	<.001	—	—	—
**Adjusted models (with covariates, including teacher-reported mental health concerns)**						
Youth-reported discipline T1 → T2/T3	3.40	[2.87, 4.03]	<.001	.490	.798	.599
Caregiver-reported discipline T1 → T2/T3	3.76	[2.97, 4.76]	<.001	—	.745	.784
Agreement across reporters T1 → T2/T3	3.54	[2.69, 4.67]	<.001	—	—	.900
Either reporter T1 → T2/T3	3.62	[3.08, 4.24]	<.001	—	—	—
**Adjusted models (with covariates, including youth-reported mental health concerns)**						
Youth-reported discipline T1 → T2/T3	3.66	[3.11, 4.31]	<.001	<.001	.012	.113
Caregiver-reported discipline T1 → T2/T3	5.92	[4.85, 7.23]	<.001	—	.545	.017
Agreement across reporters T1 → T2/T3	5.37	[4.19, 6.88]	<.001	—	—	.167
Either reporter T1 → T2/T3	4.38	[3.78, 5.08]	<.001	—	—	—

Note: N = 9,772. Unadjusted models reflect bivariate comparisons. Adjusted models with covariates included same-reporter comorbid mental health concerns, past-year school discipline, race/ethnicity, sex, grade, caregiver education, household structure, school type, and neighborhood resources. Discipline outcomes were measured as a collapsed binary variable indicating whether discipline occurred at either time 2 or time 3 (ie, during a 2-year follow-up period), whereas discipline predictors were assessed at time 1 (T1). OR = odds ratio; T = timepoint.

**Figure 2:**
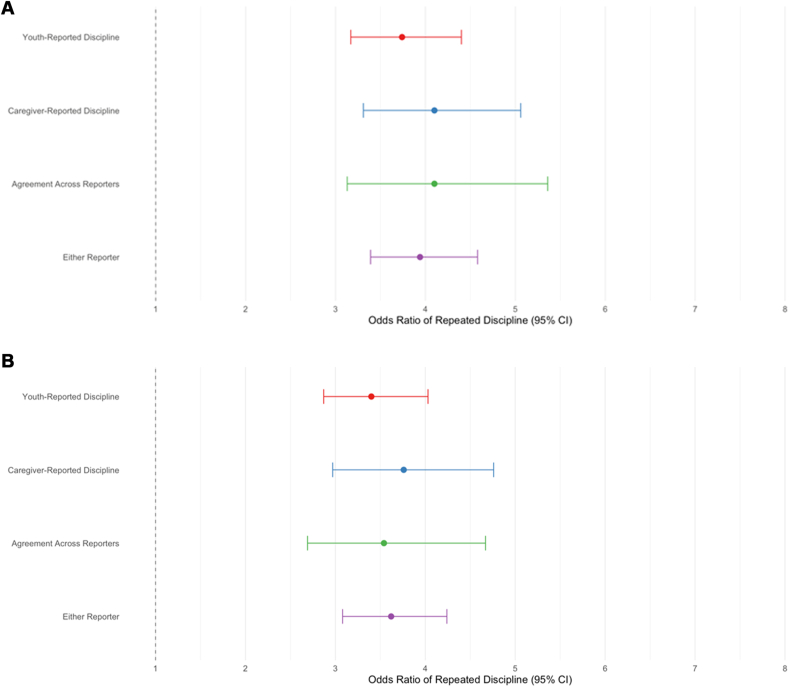

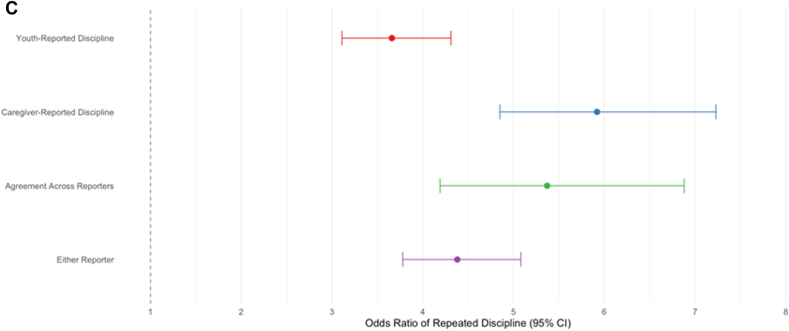
Adjusted Odds of Repeated School Discipline by Discipline Reporter and Mental Health Covariates ***Note:****(A) Controlling for caregiver-reported mental health concerns. (B) Controlling for teacher-reported mental health concerns. (C) Controlling for youth-reported mental health concerns.*

After adjusting for sociodemographics and comorbid mental health concerns, prior school discipline remained a strong predictor of subsequent discipline across all reporter groups. Effect sizes attenuated and varied depending on which set of mental health covariates was included (ORs = 3.40-5.93; *p* values < .001) (Table 3 and Figure 2). Differences in discipline risk across reporter groups were no longer significant after covariate adjustment when controlling for caregiver- or teacher-reported mental health concerns. However, when controlling for youth-reported mental health concerns, adolescents with a history of discipline were at greater risk for repeated discipline when measured via caregiver-reported discipline (*p* < .001) and agreement across reporters (*p* = .012), compared with youth-reported discipline, and when measured via caregiver-reported discipline (*p* = .017) compared to either reporter.

## Discussion

Despite significant limitations in measurement, most notably combining detentions and suspensions into a single item, the ABCD Study provides a rare opportunity to investigate a broad array of causes and consequences of school discipline. Its longitudinal design, diverse sample, comprehensive health assessments, and linkages to population-level datasets make it a valuable resource for studying developmental risk.^23^, ^24^, ^25^, ^26^ However, given that administrative records are not available, it is important to clarify how associations may differ depending on the reporter(s) used. Yet, limited guidance exists for selecting between youth- vs caregiver-reported discipline, despite the potential for each to capture distinct facets of students’ disciplinary experiences. Differences in reporting of school discipline may reflect variation in construct coverage, such as capturing more severe, salient infractions vs a broader range of minor and serious events. Although the ABCD Study’s single-item measure cannot distinguish between detentions and suspensions or capture event frequency, comparing results across reporters can still serve as a valuable sensitivity analysis, testing whether associations are robust across plausible variations in measurement rather than representing true differences in disciplinary severity.

Overall, prior externalizing and inattention/impulsivity concerns and past disciplinary history were more strongly associated with subsequent school discipline when using caregiver report or agreement across reporters than when using youth report or an “either-reporter” composite. However, differences attenuated after adjusting for covariates, suggesting that shared variance among the potential confounders, particularly when all 3 mental health concerns were entered simultaneously, accounted for many of the initial significant findings. What remained tended to be same-reporter associations, which may also reflect shared method variance in addition to shared variance with covariates. Notably, the few associations that remained significant were mostly only marginally significant.

Still, it remains important to consider what each reporter is likely capturing. Albeit speculative, caregiver-reported discipline may correspond to more severe events, whereas youth-reported discipline could encompass a broader range of infractions with weaker associations to prior behavioral problems. For example, agreement-based prior discipline was associated with 10% higher odds of subsequent discipline than youth-reported discipline, even after full adjustment. Albeit not statistically significant, this difference could become more consequential over time if compounded across multiple years. This possibility is supported by our finding that prior discipline, regardless of reporter, was a strong predictor of repeated discipline across the 2-year follow-up. Nevertheless, these distinctions should be interpreted cautiously, as the ABCD Study combines detentions and suspensions into a single measure.

Sociodemographic characteristics themselves were not consistently linked to which reporter endorsed the disciplinary event. For example, although Black and multiracial Black youth were at greater risk for experiencing discipline compared to non-Hispanic White youth, they were neither more nor less likely than their caregivers to endorse school discipline. A few exceptions emerged among Hispanic and male youth and those from higher-income households, but these patterns require replication. In contrast, sociodemographic characteristics appeared to be more influential in explaining differences across school discipline reporters in the strength of associations between mental health concerns and later discipline. Together, these findings suggest that although demographics are robustly linked to overall discipline risk, they do not fully account for why youth and their caregivers differ in whether discipline is reported.

This study has broader implications for understanding risk for school discipline. Findings highlight several key risk factors for school discipline: (1) the value of incorporating teacher reports; (2) internalizing concerns showing different associations when co-occurring behavioral problems are considered; (3) persistently elevated risk among Black and multiracial Black youth, even after accounting for externalizing concerns and prior discipline; and (4) prior discipline consistently predicting future discipline, underscoring its central role in perpetuating risk.

First, bivariate associations between mental health concerns and school discipline were largest when using teacher-reported concerns. After adjusting for covariates and other mental health concerns, teacher-reported inattention/impulsivity continued to show the largest effects on subsequent discipline. However, the use of teacher-reported data in the ABCD Study remain underused, likely because of high missingness (over 50%). Although there is no universally accepted threshold for how much missing data constitute “too much,” many researchers recommend addressing this issue through multiple imputation rather than excluding such data entirely.^27^^,^^28^ Given the strong associations between teacher-reported mental health and school discipline, our findings support that behavior observed in the school context is predictive of school-related outcomes. Accordingly, studies examining associations between adolescent behavioral health and school-related factors should include teacher-report measures as part of a multi-informant approach.

Second, the effects of internalizing concerns on school discipline reversed in direction after accounting for other behavioral concerns. In bivariate models, internalizing concerns were positively associated with risk for subsequent discipline. However, when externalizing and inattention/impulsivity concerns were added to the model, the association reversed, with higher internalizing concerns predicting a significantly lower likelihood of discipline. Moreover, youth-reported internalizing concerns no longer predicted subsequent caregiver-reported school discipline, potentially suggesting an even weaker connection between internalizing concerns and higher-severity infractions. These findings underscore the importance of considering comorbid symptom profiles when examining links between internalizing concerns and school discipline.

Third, although this study focused on individual risk factors, prevention and intervention strategies cannot target adolescent behavior alone. Consistent with prior research,^4^^,^^10^, ^11^, ^12^ Black and multiracial Black youth remained at higher risk for discipline even after accounting for behavioral concerns and prior discipline. As others have noted,^8^ interventions that do not address the disproportionate disciplining of Black youth are less effective at reducing disparities, even when they lower overall discipline rates. Thus, although behavioral concerns are strongly associated with risk of future discipline, our findings reinforce the need to address broader contextual factors. Teacher-, school-, neighborhood-, and state-level factors each play a significant role in explaining racial disparities beyond individual behavior and other demographics.^4^^,^^12^^,^^23^^,^^24^

Our findings also indicated that adolescents who received school discipline were at heightened risk for repeated infractions, independent of demographic characteristics and prior behavioral concerns. Consistent with prior studies,^4^^,^^16^^,^^25^ these results suggest that school discipline is not an effective strategy for reducing behavioral problems in schools. Instead, it may exacerbate problems by reinforcing a cycle of repeated disciplinary actions. Indeed, there is some evidence linking school discipline to long-term outcomes including mental health concerns, substance use, lower educational attainment, police contact, and financial instability.^5^, ^6^, ^7^^,^^11^^,^^26^ In addition to universal prevention efforts aimed at reducing overall discipline rates, targeted interventions for students with a history of discipline, as well as for teachers who work with them, may offer a cost-effective strategy for interrupting the cycle and decreasing reliance on exclusionary practices over time.

There are several limitations to note, which also point to important directions for future research. First and foremost, the ABCD Study was not designed to investigate the causes and consequences of school discipline, and its measurement approach constrains the inferences that can be drawn. School administrative records are widely considered the gold standard for assessing school discipline, as they provide detailed, verifiable accounts of the type, timing, and frequency of infractions. However, such records are not available in the ABCD Study. Consequently, the study relied on a single binary item administered separately to youth and caregivers, asking whether the youth had experienced a “detention or suspension” within the past year. This item does not differentiate between detentions and suspensions, quantify the number or severity of incidents, or include psychometric validation, all of which limit the precision and validity of the measure. The observed discrepancies between youth and caregiver reports may therefore reflect not only true differences in perception or severity but also measurement insensitivity inherent to the single-item design.

Future studies should seek to replicate and extend these findings using datasets that include official school records in combination with multi-item, validated instruments assessing in-school detentions, in-school and out-of-school suspensions, and expulsions. Such designs would enable a more methodologically rigorous assessment of construct validity and reporter differences. Notably, the ABCD Social Development Substudy,^11^ conducted at 5 of the 21 ABCD sites (n = 2,422), includes separate assessments of detentions and suspensions. At baseline, 162 youth enrolled in the substudy endorsed either a detention or suspension within the last 12 months; however, only 41 youth endorsed a detention but not a suspension, and an even smaller number (n = 21) endorsed a suspension but not a detention. These limited subgroup sizes, as well as the high correlations between detentions and suspensions, make it difficult to examine whether associations with mental health concerns differ by type of disciplinary action. If prevalence rates increase with age, the Social Development substudy will be better suited than the broader ABCD sample to examine these nuanced questions.

There are also likely numerous factors that drive the decision on which reporter(s) to use. We focused specifically on associations with mental health concerns and prior discipline history, given the robust literature in this area.^2^, ^3^, ^4^, ^5^, ^6^ We also compared multiple effect sizes, and most significant comparisons would not have held up under corrections for multiple comparisons. In addition, by the third wave of data collection, the COVID-19 pandemic had already begun for approximately 30% of participants. Results may vary because of changes that occurred during later time periods (eg, remote environments, COVID-related stress, transition to high school).

Finally, although we conducted a prospective study, its observational design precluded causal inference. Given our focus on clarifying how reporter choice may influence results, we did not examine bidirectional associations (eg, discipline predicting later mental health concerns), which remain an important but less established area for future research. As additional waves of ABCD Study data become publicly available, future researchers will have the opportunity to investigate the unique effects of school discipline on later adolescent and emerging adulthood outcomes, including its potential mediating and moderating roles in shaping long-term developmental trajectories across a diverse range of outcomes.

In conclusion, this study provides new insights into how school discipline is reported and associated with behavioral risk among early adolescents in the ABCD Study. Although adjusting for demographics and mental health reduced most reporter differences to non-significance, the observed patterns suggest that each reporter reflects partially distinct perspectives on disciplinary experiences. Given the study’s measurement limitations, these results should be viewed as preliminary and descriptive rather than definitive evidence of differences in the type or severity of disciplinary events captured by each source. Nonetheless, the consistently strong association between prior and future discipline underscores the persistence of disciplinary involvement and raises questions about the effectiveness of these practices. Situating these findings within the broader context of adolescent development can inform future research and policy efforts aimed at reducing discipline-related disparities.

## CRediT authorship contribution statement

**Erin L. Thompson:** Writing – review & editing, Writing – original draft, Methodology, Investigation, Funding acquisition, Formal analysis, Data curation, Conceptualization. **Ashley R. Adams:** Writing – review & editing, Investigation. **Sarah M. Lehman:** Writing – review & editing, Investigation. **Christine Kaiver:** Writing – review & editing, Investigation. **Samuel W. Hawes:** Writing – review & editing, Project administration, Data curation. **Kristin M. Scardamalia:** Writing – review & editing, Supervision. **Andy V. Pham:** Writing – review & editing, Supervision. **Raul Gonzalez:** Writing – review & editing, Supervision, Project administration, Methodology, Funding acquisition, Data curation.

## References

[1] Reed M.O., Jakubovski E., Johnson J.A., Bloch M.H. (2017). Predictors of long-term school-based behavioral outcomes in the multimodal treatment study of children with attention-deficit/hyperactivity disorder. J Child Adolesc Psychopharmacol.

[2] Cohen D.R., Lewis C., Eddy C.L. (2023). In-school and out-of-school suspension: behavioral and psychological outcomes in a predominately Black sample of middle school students. School Psych Rev.

[3] Noltemeyer A.L., Ward R.M., McLoughlin C. (2015). Relationship between school suspension and student outcomes: a meta-analysis. School Psych Rev.

[4] Welsh R.O., Little S. (2018). The school discipline dilemma: a comprehensive review of disparities and alternative approaches. Rev Educ Res.

[5] Mittleman J. (2018). A downward spiral? Childhood suspension and the path to juvenile arrest. Sociol Educ.

[6] Welsh R.O., Little S. (2018). Caste and control in schools: a systematic review of the pathways, rates and correlates of exclusion due to school discipline. Child Youth Serv Rev.

[7] Duarte C d. P, Moses C., Brown M. (2022). Punitive school discipline as a mechanism of structural marginalization with implications for health inequity: a systematic review of quantitative studies in the health and social sciences literature. Ann N Y Acad Sci.

[8] Gregory A., Osher D., Bear G.G., Jagers R.J., Sprague J.R. (2021). Good intentions are not enough: centering equity in school discipline reform. School Psych Rev.

[9] Jernigan T.L., Brown S.A., Dowling G.J. (2018). The adolescent brain cognitive development study. J Res Adolesc.

[10] Fadus M.C., Valadez E.A., Bryant B.E. (2021). Racial disparities in elementary school disciplinary actions: findings from the ABCD Study. J Am Acad Child Adolesc Psychiatry.

[11] Brislin S.J., Choi M., Perkins E.R. (2024). Racial bias in school discipline and police contact: evidence from the Adolescent Brain Cognitive Development Social Development (ABCD-SD) Study. J Am Acad Child Adolesc Psychiatry.

[12] Thompson E.L., Gonzalez M.R., Scardamalia K.M. (2025). Structural determinants of school discipline: examining state-level racial bias and neighborhood opportunity. J Am Acad Child Adolesc Psychiatry.

[13] Assari S., Zare H. (2024). Black-White gap across levels of educational childhood opportunities: findings from the ABCD Study. Open J Educ Res.

[14] De Los Reyes A. (2024).

[15] Public Counsel (2021). Parent guidebook on school discipline, Public Counsel. http://www.fixschooldiscipline.org/wp-content/uploads/2021/10/Final.Final_.ENGLISH.Parent.Guidebook.School.Discipline.pdf.

[16] Garavan H., Bartsch H., Conway K. (2018). Recruiting the ABCD sample: design considerations and procedures. Dev Cogn Neurosci.

[17] LiCalsi C., D. Osher, P. Bailey (2021). http://www.air.org/sites/default/files/2021-08/NYC-Suspension-Effects-Behavioral-Academic-Outcomes-August-2021.pdf.

[18] Heeringa S.G., Berglund P.A. (Published online February 10, 2020). A guide for population-based analysis of the Adolescent Brain Cognitive Development (ABCD) Study baseline data. bioRxiv.

[19] Achenbach T.M., Rescorla L.A. (2001).

[20] Achenbach T.M., McConaughy S.H., Ivanova M.Y., Rescorla L.A. (2011). Manual for the ASEBA Brief Problem Monitor for Ages 6–18 (BPM/6–18).

[21] Acevedo-Garcia D., Noelke C., McArdle N. (2020). Racial and ethnic inequities in children’s neighborhoods: evidence from the New Child Opportunity Index 2.0. Health Aff.

[22] Audigier V., White I.R., Jolani S. (2017). Multiple imputation for multilevel data with continuous and binary variables. Stat Sci.

[23] Owens J. (2022). Double jeopardy: teacher biases, racialized organizations, and the production of racial/ethnic disparities in school discipline. Am Sociol Rev.

[24] Okonofua J.A., Walton G.M., Eberhardt J.L. (2016). A vicious cycle: a social-psychological account of extreme racial disparities in school discipline. Persp Psychol Sci.

[25] Gerlinger J., Viano S., Gardella J.H., Fisher B.W., Chris Curran F., Higgins E.M. (2021). Exclusionary school discipline and delinquent outcomes: a meta-analysis. J Youth Adolesc.

[26] Rosenbaum J. (2020). Educational and criminal justice outcomes 12 years after school suspension. Youth Soc.

[27] Hayes T., Enders C.K., Cooper H., Panter A., Rindskopf D., Sher K., Coutanche M., McMullen L. (2023).

[28] Li L., Bayat M., Hayes T.B. (Published online June 13, 2024). Missing data approaches for longitudinal neuroimaging research: examples from the Adolescent Brain Cognitive Development (ABCD) Study. bioRxiv.

