# Supplementary material for: Reporter Discrepancies in the Associations Between Mental Health Concerns and School Discipline
Source: JAACAP Open. 2026 Jan 13;4(3):475–87. doi: 10.1016/j.jaacop.2026.01.001 (PMC13221827; doi:10.1016/j.jaacop.2026.01.001)
Supplement: Supplementary Tables S1-S5 [file mmc1.docx]

Reporter Discrepancies in the Associations Between Mental Health Concerns and School Discipline

**Table S1.** Demographic Characteristics of the ABCD Study Sample (*n* = 9,772)

|  | **T0** | **T1** | **T2** | **T3** |
| --- | --- | --- | --- | --- |
| **Youth-Reported School Discipline** |  |  |  |  |
| No | 8,837 (90.43) | 8,313 (85.07) | 7,806 (79.88) | 7,405 (75.78) |
| Yes | 921 (9.42) | 878 (8.98) | 1,172 (11.99) | 1,003 (10.26) |
| Missing | 14 (0.14) | 581 (5.95) | 794 (8.13) | 1,364 (13.96) |
| **Caregiver-Reported School Discipline** |  |  |  |  |
| No | 9,058 (92.69) | 8,371 (85.66) | 7,896 (80.80) | 7,272 (74.42) |
| Yes | 549 (5.61) | 622 (6.37) | 722 (7.39) | 540 (5.53) |
| Missing | 165 (1.69) | 779 (7.97) | 1,154 (11.81) | 1,960 (20.06) |
|  | **T0 Predictors** | |  |  |
| **Sex assigned at birth** |  |  |  |  |
| Male^a^ | 5,125 (52.45) | |  |  |
| Female | 4,647 (47.55) | |  |  |
| Missing | 0.00 |  |  |  |
| **Two-caregiver household** |  |  |  |  |
| No | 1,838 (18.81) | |  |  |
| Yes | 7,274 (74.44) | |  |  |
| Missing | 660 (6.75) |  |  |  |
| **Neighborhood opportunity (SD)** |  |  |  |  |
| Very Low Opportunities | 1,585 (16.22) | |  |  |
| Low Opportunities | 1,106 (11.32) | |  |  |
| Medium Opportunities | 1,272 (13.02) | |  |  |
| High Opportunities | 2,073 (21.21) | |  |  |
| Very High Opportunities | 2,931 (29.99) | |  |  |
| Missing | 805 (8.23) |  |  |  |
| **Race and ethnicity** |  |  |  |  |
| Black (Non-Hispanic) | 1,484 (15.19) | |  |  |
| Multiracial Black | 606 (6.20) | |  |  |
| Hispanic (Non-Black) | 1,901 (19.45) | |  |  |
| Other (Non-Hispanic/Non-Black)^b^ | 826 (8.45) |  |  |  |
| White (Non-Hispanic/Non-Black) | 4,947(50.62) | |  |  |
| Missing | 8 (0.08) |  |  |  |
| **Caregiver education** |  |  |  |  |
| Less than high school diploma | 515 (5.27) |  |  |  |
| HS diploma/GED | 1,221 (12.49) | |  |  |
| Some college | 2,865 (29.32) | |  |  |
| Bachelor’s degree | 2,684 (27.47) | |  |  |
| Post-Bachelor’s degree | 2,473 (25.31) | |  |  |
| Missing | 14 (0.14) |  |  |  |
|  |  | **T1 Predictors** | |  |
| **Mental Health Concerns** |  |  |  |  |
| TR-Externalizing Mean (SD) |  | 1.29 (2.38) |  |  |
| Missing |  | 5,693 (58.26) | |  |
| TR-Inattention/Impulsivity Mean (SD) |  | 2.85 (3.25) |  |  |
| Missing |  | 6,490 (66.41) | |  |
| TR-Internalizing Mean (SD) |  | 1.73 (2.30) |  |  |
| Missing |  | 5,692 (58.24) | |  |
| CR-Externalizing Mean (SD) |  | 4.26 (5.68) |  |  |
| Missing |  | 578 (6.26) |  |  |
| CR-Inattention/Impulsivity Mean (SD) |  | 2.97 (3.48) |  |  |
| Missing |  | 578 (6.26) |  |  |
| CR-Internalizing Mean (SD) |  | 5.30 (5.67) |  |  |
| Missing |  | 578 (6.26) |  |  |
|  |  | **T1 Predictors** | |  |
| YR-Externalizing Mean (SD) |  | 1.97 (2.01) |  |  |
| Missing |  | 1,196 (12.24) | |  |
| YR-Inattention/Impulsivity Mean (SD) |  | 3.21 (2.67) |  |  |
| Missing |  | 1,468 (15.02) | |  |
| YR-Internalizing Mean (SD) |  | 1.76 (2.13) |  |  |
| Missing |  | 1,104 (11.30) | |  |
| **Grade** |  |  |  |  |
| 4^th^ |  | 1,690 (17.29) | |  |
| 5^th^ |  | 4,156 (42.53) | |  |
| 6^th^ |  | 3,052 (31.23) | |  |
| 7^th^ |  | 300 (3.38) |  |  |
| Missing |  | 574 (5.87) |  |  |
| **Age** |  |  |  |  |
| 9 |  | 372 (3.81) |  |  |
| 10 |  | 4,522 (46.28) | |  |
| 11 |  | 4,006 (40.99) | |  |
| 12 |  | 308 (3.15) |  |  |
| Missing |  | 564 (5.77) |  |  |
| **School type** |  |  |  |  |
| Attend public school |  | 7,613 (77.91) | |  |
| Attend private school |  | 483 (4.94) |  |  |
| Attend charter school |  | 680 (6.96) |  |  |
| Attend other type of school |  | 414 (4.24) |  |  |
| Missing |  | 582 (5.96) |  |  |
| **Household income** |  |  |  |  |
| Under $50,000 |  | 2,380 (24.36) | |  |
| $50,000 - $99,999 |  | 2,313 (23.67) | |  |
| $100,000 and over |  | 3,812 (39.01) | |  |
| Missing |  | 1,267 (12.97) | |  |

*Note.* We randomly sampled one member from each family. Total ABCD Study sample is 11,876. Distributions are represented as *n* (%) unless otherwise noted. Only variables included in the imputation model are presented. ^a^Less than 10 caregivers endorsed “Intersex-Male.” ^b^The “Other race(s)” category includes youth whose caregiver identified them as American Indian, Native American, Alaska Native, Native Hawaiian, Guamanian, Samoan, Other Pacific Islander, Asian Indian, Chinese, Filipino, Japanese, Korean, Vietnamese, Other Asian, Other Race, or as belonging to more than one race (excluding African American or Black youth). These were recoded as male. SD = Standard Deviation. TR = Teacher-Reported. CR = Caregiver-Reported. YR = Youth-Reported.

**Table S2.** Bivariate (Unadjusted) Associations Between Mental Health Concerns and School Discipline Across Reporters

|  | Outcomes | | | | | | | | | | | |  |
| --- | --- | --- | --- | --- | --- | --- | --- | --- | --- | --- | --- | --- | --- |
|  | A | |  | B | |  | C | |  | D | |  |  |
|  | *Youth-Reported Discipline* | | | *Caregiver-Reported Discipline* | | | *Agreement Across Reporters* | | | *Either Reporter* | | |  |
| Predictors | OR | 95%  OR CI | *p* | OR | 95%  OR CI | *p* | OR | 95%  OR CI | *p* | OR | 95%  OR CI | *p* | Comp^a^  *p* < .05 |
| **Externalizing Concerns** | |  |  |  |  |  |  |  |  |  |  |  |  |
| Caregiver-reported | 1.57 | [1.49,1.65] | <.001 | 1.88 | [1.77,1.99] | <.001 | 1.78 | [1.67,1.89] | <.001 | 1.72 | [1.64,1.81] | <.001 | B, C, D > A  B > D |
| Teacher-reported | 1.72 | [1.62,1.83] | <.001 | 1.92 | [1.81,2.05] | <.001 | 1.90 | [1.78,2.03] | <.001 | 1.85 | [1.73,1.97] | <.001 | B, C, D > A |
| Youth-reported | 1.56 | [1.48,1.65] | <.001 | 1.55 | [1.46,1.64] | <.001 | 1.65 | [1.54,1.76] | <.001 | 1.55 | [1.48,1.63] | <.001 | None |
| **Inattention/Impulsivity Concerns** | | |  |  |  |  |  |  |  |  |  |  |  |
| Caregiver-reported | 1.42 | [1.35,1.49] | <.001 | 1.65 | [1.56,1.75] | <.001 | 1.59 | [1.50,1.69] | <.001 | 1.51 | [1.44,1.59] | <.001 | B, C, D > A  B > D |
| Teacher-reported | 1.79 | [1.68,1.90] | <.001 | 1.98 | [1.85,2.12] | <.001 | 2.02 | [1.86,2.19] | <.001 | 1.86 | [1.75,1.97] | <.001 | B, C > A |
| Youth-reported | 1.56 | [1.48,1.64] | <.001 | 1.44 | [1.35,1.54] | <.001 | 1.56 | [1.45,1.68] | <.001 | 1.51 | [1.43,1.59] | <.001 | None |
| **Internalizing Concerns** | | |  |  |  |  |  |  |  |  |  |  |  |
| Caregiver-reported | 1.08 | [1.03,1.14] | .002 | 1.21 | [1.14,1.29] | <.001 | 1.19 | [1.11,1.27] | <.001 | 1.12 | [1.07,1.17] | <.001 | B, C > A  B > D |
| Teacher-reported | 1.22 | [1.15,1.29] | <.001 | 1.29 | [1.21,1.38] | <.001 | 1.30 | [1.21,1.39] | <.001 | 1.24 | [1.17,1.31] | <.001 | None |
| Youth-reported | 1.19 | [1.13,1.26] | <.001 | 1.17 | [1.11,1.24] | <.001 | 1.21 | [1.13,1.30] | <.001 | 1.18 | [1.12,1.24] | <.001 | None |

Note. *n* = 9,772. All associations are unadjusted and reflect bivariate comparisons. Outcomes were measured across Time 2 and Time 3, whereas predictors were assessed at T1. OR = Odds Ratio. CI = Confidence Interval. TR = Teacher-Reported. CR = Caregiver-Reported. YR = Youth-Reported. Comp = Comparison. ^a^Comparisons reflect results from z-tests evaluating whether the effect of a given predictor differed across regression models with different outcomes. Directional differences (e.g., B > A) indicate a statistically significant difference in effect size between school discipline reporters at *p* < .05.

**Table S3.** Associations Between Caregiver-Reported Mental Health Concerns and School Discipline Across Reporters After Accounting for Covariates

|  | Outcomes | | | | | | | |  |
| --- | --- | --- | --- | --- | --- | --- | --- | --- | --- |
|  | A | | B | | C | | D | |  |
|  | *Youth-Reported Discipline* | | *Caregiver-Reported Discipline* | | *Agreement Across Reporters* | | *Either Reporter* | | Comparisons  *p* < .05 |
| Predictors | OR | 95% OR CI | OR | 95% OR CI | OR | 95% OR CI | OR | 95% OR CI |  |
| CR-externalizing concerns | 1.53 | [1.40,1.66] | 1.75 | [1.60,1.91] | 1.73 | [1.56,1.92] | 1.63 | [1.51,1.77] | B > A |
| CR-inattention/impulsivity concerns | 1.15 | [1.07,1.24] | 1.18 | [1.08,1.29] | 1.15 | [1.05,1.27] | 1.18 | [1.10,1.27] | None |
| CR-internalizing concerns | 0.75 | [0.70,0.81] | 0.73 | [0.67,0.80] | 0.73 | [0.66,0.81] | 0.74 | [0.68,0.79] | None |
| Prior year discipline^a^ | 3.74 | [3.18,4.41] | 4.14 | [3.35,5.11] | 4.13 | [3.16,5.39] | 3.96 | [3.41,4.60] | - |
| Black (Non-Hispanic)^b^ | 1.55 | [1.27,1.90] | 1.52 | [1.18,1.95] | 1.51 | [1.14,1.99] | 1.60 | [1.32,1.94] | - |
| Hispanic (Non-Black) ^b^ | 1.21 | [1.00,1.47] | 1.13 | [0.88,1.44] | 1.20 | [0.92,1.57] | 1.18 | [0.98,1.42] | - |
| Other (Non-Hispanic/Non-Black) ^b^ | 0.93 | [0.73,1.19] | 1.01 | [0.75,1.36] | 0.95 | [0.67,1.33] | 0.97 | [0.77,1.23] | - |
| Multiracial Black ^b^ | 1.52 | [1.20,1.92] | 1.61 | [1.19,2.17] | 1.62 | [1.18,2.23] | 1.57 | [1.24,1.98] | - |
| Male^c^ | 1.44 | [1.28,1.61] | 1.65 | [1.42,1.92] | 1.56 | [1.32,1.85] | 1.52 | [1.36,1.71] | - |
| Caregiver education | 0.83 | [0.78,0.89] | 0.80 | [0.74,0.87] | 0.81 | [0.74,0.88] | 0.82 | [0.77,0.87] | - |
| Two-caregiver household | 0.85 | [0.73,0.99] | 0.83 | [0.70,0.99] | 0.80 | [0.66,0.96] | 0.86 | [0.73,1.00] | - |
| Attend private school^d^ | 1.00 | [0.76,1.31] | 0.96 | [0.68,1.34] | 0.96 | [0.66,1.40] | 0.99 | [0.76,1.30] | - |
| Attend charter school^d^ | 1.25 | [1.00,1.56] | 1.51 | [1.17,1.95] | 1.53 | [1.16,2.02] | 1.29 | [1.03,1.60] | - |
| Attend other type of school^d^ | 0.58 | [0.41,0.82] | 0.75 | [0.50,1.13] | 0.64 | [0.39,1.05] | 0.63 | [0.45,0.87] | - |
| Grade | 1.44 | [1.33,1.57] | 1.32 | [1.20,1.45] | 1.38 | [1.24,1.54] | 1.42 | [1.31,1.54] | - |
| Low opportunity neighborhood^e^ | 1.14 | [0.92,1.41] | 1.17 | [0.90,1.53] | 1.09 | [0.81,1.46] | 1.20 | [0.98,1.47] | - |
| Medium opportunity neighborhood^e^ | 0.97 | [0.78,1.22] | 1.44 | [1.10,1.88] | 1.29 | [0.96,1.73] | 1.08 | [0.87,1.34] | - |
| High opportunity neighborhood^e^ | 1.00 | [0.80,1.24] | 1.44 | [1.11,1.87] | 1.32 | [1.00,1.76] | 1.10 | [0.89,1.36] | - |
| Very high opportunity neighborhood^e^ | 0.80 | [0.63,1.01] | 1.12 | [0.85,1.47] | 0.98 | [0.72,1.32] | 0.88 | [0.70,1.11] | - |

*Note*. *n* = 9,772. CR = Caregiver-report. ^a^Prior-year discipline corresponded to the same reporter(s) used in the outcome (e.g., Prior year caregiver-reported discipline when the outcome was also caregiver-reported discipline). ^b^Non-Hispanic White youth were the reference group. ^c^Females were the reference group. ^d^Attending public school was the reference group. ^e^Very low opportunity neighborhoods were the reference group.

**Table S4.** Associations Between Teacher-Reported Mental Health Concerns and School Discipline Across Reporters After Accounting for Covariates

|  | Outcomes | | | | | | | |  |
| --- | --- | --- | --- | --- | --- | --- | --- | --- | --- |
|  | A | | B | | C | | D | |  |
|  | *Youth-Reported Discipline* | | *Caregiver-Reported Discipline* | | *Agreement Across Reporters* | | *Either Reporter* | | Comparisons  *p* < .05 |
| Predictors | OR | 95% OR CI | OR | 95% OR CI | OR | 95% OR CI | OR | 95% OR CI |  |
| TR-externalizing concerns | 1.33 | [1.22,1.45] | 1.44 | [1.30,1.60] | 1.49 | [1.34,1.65] | 1.37 | [1.25,1.49] | None |
| TR-inattention/impulsivity concerns | 1.36 | [1.26,1.48] | 1.36 | [1.21,1.53] | 1.41 | [1.24,1.60] | 1.36 | [1.26,1.48] | None |
| TR-internalizing concerns | 0.90 | [0.84,0.97] | 0.91 | [0.83,0.99] | 0.89 | [0.81,0.98] | 0.90 | [0.84,0.97] | None |
| Prior year discipline^a^ | 3.40 | [2.87,4.03] | 3.76 | [2.97,4.76] | 3.54 | [2.69,4.67] | 3.62 | [3.08,4.24] | - |
| Black (Non-Hispanic)^b^ | 1.29 | [1.04,1.59] | 1.16 | [0.90,1.50] | 1.11 | [0.82,1.50] | 1.32 | [1.08,1.61] | - |
| Hispanic (Non-Black) ^b^ | 1.13 | [0.93,1.38] | 1.03 | [0.81,1.32] | 1.09 | [0.83,1.43] | 1.10 | [0.91,1.32] | - |
| Other (Non-Hispanic/Non-Black) ^b^ | 0.92 | [0.72,1.17] | 0.97 | [0.71,1.31] | 0.91 | [0.65,1.28] | 0.95 | [0.75,1.20] | - |
| Multiracial Black ^b^ | 1.34 | [1.05,1.70] | 1.35 | [1.00,1.83] | 1.32 | [0.96,1.84] | 1.38 | [1.09,1.74] | - |
| Male^c^ | 1.26 | [1.12,1.43] | 1.44 | [1.23,1.69] | 1.31 | [1.10,1.56] | 1.36 | [1.21,1.53] | - |
| Caregiver education | 0.86 | [0.80,0.92] | 0.83 | [0.77,0.90] | 0.84 | [0.77,0.92] | 0.84 | [0.79,0.90] | - |
| Two-caregiver household | 0.92 | [0.79,1.09] | 0.90 | [0.75,1.08] | 0.88 | [0.72,1.08] | 0.93 | [0.79,1.08] | - |
| Attend private school^d^ | 0.93 | [0.71,1.23] | 0.85 | [0.60,1.21] | 0.85 | [0.58,1.26] | 0.91 | [0.69,1.20] | - |
| Attend charter school^d^ | 1.23 | [0.98,1.54] | 1.49 | [1.15,1.93] | 1.51 | [1.14,2.00] | 1.26 | [1.01,1.57] | - |
| Attend other type of school^d^ | 0.54 | [0.38,0.76] | 0.72 | [0.48,1.07] | 0.60 | [0.37,0.98] | 0.59 | [0.43,0.81] | - |
| Grade | 1.45 | [1.33,1.58] | 1.31 | [1.18,1.45] | 1.39 | [1.23,1.56] | 1.42 | [1.31,1.55] | - |
| Low opportunity neighborhood^e^ | 1.14 | [0.92,1.40] | 1.18 | [0.91,1.53] | 1.09 | [0.82,1.46] | 1.20 | [0.98,1.46] | - |
| Medium opportunity neighborhood^e^ | 0.97 | [0.77,1.23] | 1.46 | [1.11,1.92] | 1.33 | [0.98,1.81] | 1.07 | [0.86,1.33] | - |
| High opportunity neighborhood^e^ | 0.95 | [0.75,1.19] | 1.38 | [1.05,1.81] | 1.26 | [0.93,1.71] | 1.04 | [0.84,1.29] | - |
| Very high opportunity neighborhood^e^ | 0.76 | [0.60,0.97] | 1.09 | [0.82,1.43] | 0.96 | [0.70,1.31] | 0.84 | [0.67,1.06] | - |

*Note*. *n* = 9,772. TR = Teacher-report. ^a^Prior-year discipline corresponded to the same reporter(s) used in the outcome (e.g., Prior year caregiver-reported discipline when the outcome was also caregiver-reported discipline). ^b^Non-Hispanic White youth were the reference group. ^c^Females were the reference group. ^d^Attending public school was the reference group. ^e^Very low opportunity neighborhoods were the reference group.

**Table S5.** Associations Between Youth-Reported Mental Health Concerns and School Discipline Across Reporters After Accounting for Covariates

|  | Outcomes | | | | | | | |  |
| --- | --- | --- | --- | --- | --- | --- | --- | --- | --- |
|  | A | | B | | C | | D | |  |
|  | Youth-Reported Discipline | | Caregiver-Reported Discipline | | Agreement Across Reporters | | Either Reporter | | Comparisons  *p* < .05 |
| Predictors | OR | 95% OR CI | OR | 95% OR CI | OR | 95% OR CI | OR | 95% OR CI |  |
| YR-externalizing concerns | 1.27 | [1.18,1.36] | 1.32 | [1.21,1.44] | 1.38 | [1.26,1.52] | 1.25 | [1.17,1.34] | None |
| YR-inattention/impulsivity concerns | 1.27 | [1.18,1.37] | 1.11 | [1.00,1.22] | 1.18 | [1.06,1.31] | 1.22 | [1.13,1.31] | A > B |
| YR-internalizing concerns | 0.90 | [0.83,0.97] | 0.95 | [0.87,1.04] | 0.92 | [0.84,1.02] | 0.91 | [0.84,0.98] | None |
| Prior year discipline^a^ | 3.66 | [3.11,4.31] | 5.92 | [4.85,7.23] | 5.37 | [4.19,6.88] | 4.38 | [3.78,5.08] | - |
| Black (Non-Hispanic)^b^ | 1.54 | [1.26,1.88] | 1.43 | [1.12,1.82] | 1.45 | [1.10,1.90] | 1.56 | [1.29,1.89] | - |
| Hispanic (Non-Black) ^b^ | 1.16 | [0.96,1.40] | 1.05 | [0.83,1.34] | 1.12 | [0.86,1.45] | 1.12 | [0.93,1.34] | - |
| Other (Non-Hispanic/Non-Black) ^b^ | 0.90 | [0.70,1.14] | 0.93 | [0.69,1.25] | 0.87 | [0.62,1.22] | 0.92 | [0.73,1.16] | - |
| Multiracial Black ^b^ | 1.47 | [1.16,1.85] | 1.54 | [1.15,2.06] | 1.55 | [1.14,2.12] | 1.50 | [1.20,1.89] | - |
| Male^c^ | 1.53 | [1.36,1.71] | 1.80 | [1.55,2.09] | 1.70 | [1.43,2.01] | 1.63 | [1.46,1.82] | - |
| Caregiver education | 0.84 | [0.79,0.89] | 0.80 | [0.75,0.87] | 0.82 | [0.75,0.89] | 0.82 | [0.77,0.87] | - |
| Two-caregiver household | 0.83 | [0.71,0.97] | 0.80 | [0.68,0.94] | 0.77 | [0.64,0.92] | 0.83 | [0.72,0.96] | - |
| Attend private school^d^ | 0.97 | [0.74,1.27] | 0.88 | [0.63,1.23] | 0.89 | [0.61,1.29] | 0.95 | [0.73,1.23] | - |
| Attend charter school^d^ | 1.25 | [1.00,1.56] | 1.49 | [1.16,1.92] | 1.51 | [1.15,2.00] | 1.28 | [1.02,1.59] | - |
| Attend other type of school^d^ | 0.56 | [0.40,0.80] | 0.80 | [0.54,1.18] | 0.67 | [0.42,1.09] | 0.63 | [0.46,0.87] | - |
| Grade | 1.40 | [1.29,1.52] | 1.24 | [1.13,1.37] | 1.31 | [1.18,1.47] | 1.36 | [1.26,1.47] | - |
| Low opportunity neighborhood^e^ | 1.07 | [0.87,1.32] | 1.10 | [0.84,1.42] | 1.00 | [0.75,1.34] | 1.13 | [0.93,1.39] | - |
| Medium opportunity neighborhood^e^ | 0.89 | [0.72,1.11] | 1.30 | [1.00,1.69] | 1.15 | [0.86,1.54] | 1.00 | [0.81,1.23] | - |
| High opportunity neighborhood^e^ | 0.94 | [0.75,1.16] | 1.31 | [1.02,1.70] | 1.19 | [0.90,1.58] | 1.04 | [0.84,1.28] | - |
| Very high opportunity neighborhood^e^ | 0.78 | [0.62,0.98] | 1.07 | [0.82,1.39] | 0.93 | [0.69,1.24] | 0.86 | [0.69,1.08] | - |

*Note*. *n* = 9,772. YR = Youth-report. ^a^Prior-year discipline corresponded to the same reporter(s) used in the outcome (e.g., Prior year caregiver-reported discipline when the outcome was also caregiver-reported discipline). ^b^Non-Hispanic White youth were the reference group. ^c^Females were the reference group. ^d^Attending public school was the reference group. ^e^Very low opportunity neighborhoods were the reference group.
